# Genetic biomarkers in osteoarthritis: a quick overview

**DOI:** 10.12703/r/10-78

**Published:** 2021-11-10

**Authors:** Ignacio Rego-Pérez, Alejandro Durán-Sotuela, Paula Ramos-Louro, Francisco J Blanco

**Affiliations:** 1 Unidad de Genómica. Grupo de Investigación en Reumatología (GIR). Instituto de Investigación Biomédica de A Coruña (INIBIC), Complexo Hospitalario Universitario de A Coruña (CHUAC), Sergas. Universidade da Coruña (UDC). C/ As Xubias de Arriba 84, 15006, A Coruña, España; 2 Universidade da Coruña (UDC), Grupo de Investigación en Reumatología y Salud. Departamento de Fisioterapia, Medicina y Ciencias Biomédicas, Facultad de Fisioterapia, Campus de Oza, 15008, A Coruña, España

**Keywords:** Osteoarthritis, Biomarkers, Genetics

## Abstract

Osteoarthritis (OA) is a chronic musculoskeletal disease with a polygenic and heterogeneous nature. In addition, when clinical manifestations appear, the evolution of the disease is usually already irreversible. Therefore, the efforts on OA research are focused mainly on the discovery of therapeutic targets and reliable biomarkers that permit the early identification of different OA-related parameters such as diagnosis, prognosis, or phenotype identification. To date, potential candidate protein biomarkers have been associated with different aspects of the disease; however, there is currently no gold standard. In this sense, genomic data could act as complementary biomarkers of diagnosis and prognosis or even help to identify therapeutic targets of the disease. In this review, we will describe the most recent advances in genetic biomarkers in OA over the past three years.

Genetic biomarkers in osteoarthritis (OA) could serve as potential indicators for various disease parameters, including diagnosis or prognosis, and even act as surrogate endpoints in clinical trials for disease-modifying drugs. Thanks to recent technological advances, it has been possible to analyze data from single-cell RNA sequencing (RNA-Seq), non-coding RNAs, genome-wide DNA methylation, or genome-wide association studies (GWASs). These advances, combined with online databases and high-throughput platforms, permitted investigators to gather an unprecedented amount of genomic data in OA research.

## Single-cell RNA sequencing

Although many challenges remain, substantial progress has been made in recent years in obtaining high-quality single-cell data sequencing, resulting in the discovery of unexpected new biology and leading to new insights into development and disease^1^. In the field of OA, the first human cartilage single-cell RNA-Seq was performed by Quanbo Ji *et al*.^2^, who analyzed the transcriptome of 1464 isolated chondrocytes from 10 patients undergoing knee arthroplasty surgery. The authors identified seven molecularly defined populations of chondrocytes, three of which consisted of novel phenotypes with distinct functions, termed homeostatic chondrocytes, regulatory chondrocytes, and effector chondrocytes. Differential expression analyses between these phenotypes revealed newly identified OA susceptibility regions. Subsequent gene expression assays at both the tissue and cellular levels concluded that *COL6A3* and *ACTG1* genes might participate in the progression of OA^3^. Another recent study aimed to elucidate the molecular cross-talk between cartilage and synovium by using single-cell RNA-Seq in matched cartilage and synovium from patients with OA and concluded that most of the key OA-related cytokines were produced by synoviocytes but that only a smaller proportion was produced by chondrocytes; interestingly, none of these cytokines was exclusively expressed by chondrocytes^4^. In addition, these authors revealed the presence of a distinct subset of *HLA-DRA^+^* synoviocytes, characterized by the high expression of classic OA-related pro-inflammatory cytokines, including *IL1B, IL1A, IL6, TNF, CCL2*, and *CCL3*^4^. The authors propose to target these cell populations as an opportunity for early therapeutic interventions in OA.

## microRNAs

microRNAs (miRNAs) are small non-coding RNAs of 18 to 28 nucleotides and function as major players of post-transcriptional regulation of protein expression by binding specific complementary sequences in the 3′-untranslated region of messenger RNAs (mRNAs) to negatively regulate gene expression^5^. Their involvement in normal and pathological processes has been demonstrated. Studies performed during the previous decade in OA included the identification of candidate miRNAs for miRNA-based OA therapy as well as their usefulness as biomarkers of disease^6–8^. More recently, Coutinho de Almeida *et al*. performed an integration approach by genome-wide RNA-Seq of paired mRNA and miRNAs on macroscopically preserved and lesioned OA cartilage from the same patient to build an OA-specific miRNA interactome. The authors found 142 miRNAs and 2387 mRNAs differentially expressed between lesioned and preserved OA cartilage as well as a regulatory network of 62 miRNAs targeting up to 238 mRNAs. Among their findings, the miR-99a-3p miRNA, not previously associated with OA, stands out. This miRNA targets the highest number of genes, including *FZD1* or *GDF6*^9^.

Interesting results have also been revealed by analyzing the miR-34a-5p. This miRNA, whose expression is increased in articular cartilage, plasma, and synovial fluid of patients with late knee OA (Kellgren–Lawrence [KL] grade III and IV) compared with early radiographic knee OA (KL grade 0 and I) and even in obese patients with late knee OA compared with non-obese patients with late knee OA, promotes the expression of key catabolic markers and reduces that of extracellular matrix markers in human OA chondrocytes^10^. Later, the role of this miRNA in OA pathophysiology was supported by the fact that miR-34a-KO mice subjected to destabilization of the medial meniscus (DMM) surgery exhibited protection from cartilage degeneration^10^. The authors conclude that the joint destructive effects of this miRNA could be attenuated by using antisense oligonucleotide (ASO) technology.

Another recent study, based on in-depth sequencing of plasma circulating miRNAs, also proposed miRNAs as biomarkers of early symptomatic radiographic knee OA^11^. The authors identified a set of 97 miRNAs with a significant differential expression in at least 85% of samples in the early OA group (n = 41 subjects with KL grade 0 or I), compared with the median expression in the late OA group (n = 50 subjects with KL grade III or IV), of which seven (hsa-miR-335-3p, hsa-miR-199a-5p, hsa-miR-671-3p, hsa-miR-1260b, has-miR-191-3p, hsa-miR-335-5p, and hsa-miR-543) remained significant when the threshold was increased to at least 95% of samples. In addition, the authors identified four novel miRNAs. Their computational approaches revealed 27 common gene targets, including several matrix metalloproteinases, aggrecanases, and collagens, contributing to cartilage catabolism and anabolism^11^. The use of next-generation sequencing techniques applied to the analysis of circulating miRNAs was also used to assess their association with prevalent and incident knee OA in women. In this sense, Jean-Charles Rousseau *et al*. revealed that circulating miR-146a-5p and miR-186-5p are significantly associated with prevalent and incident knee OA, respectively, in women^12^.

Several miRNAs are involved in the modulation of autophagy. Autophagy is a necessary process to maintain cellular homeostasis in articular cartilage, reduce chondrocyte apoptosis, and inhibit inflammation; defective autophagy plays an important role in the development of OA^13^. Other studies demonstrated the role of several miRNAs in the modulation of autophagy in OA^14–16^. Specifically, circulating miRNA let-7e levels have been reported to be decreased in patients with knee OA and were associated with an increased risk of arthroplasty due to severe OA^6^. More recently, Feng *et al*. examined the expression of circulating miR-let-7e in 10 patients with knee OA and 10 subjects with trauma without knee OA, followed by the expression of this miRNA in cartilage and serum of a knee OA rat model. In addition, they examined the expression of apoptotic and autophagy-related proteins in cartilage. They demonstrated that, compared with controls, miR-let-7e levels were significantly decreased in peripheral serum of patients with knee OA, as well as in serum and cartilage of rats with knee OA, together with an increased expression of proteins involved in the apoptotic pathway and decreased expression of autophagy-related proteins. Interestingly, the authors conclude that these effects may be reversed by restoring the expression of miR-let-7e^17^.

## Long non-coding RNAs

Long non-coding RNAs (lncRNAs) are a group of non-coding RNAs of more than 200 nucleotides in length^18^. lncRNAs are able to modulate mRNA stability, translation, and post-translational modification in the cytoplasm^19^. Recent studies have pointed to a regulatory role related to the maintenance and progression of inflammatory diseases. In the case of OA, lncRNAs regulate chondrocyte proliferation, apoptosis, chondrocyte-related synovitis, and chondrocyte-associated extracellular matrix degradation, suggesting that these molecules are essential regulators of cartilage degeneration during OA. Actually, they are considered potential diagnostic biomarkers and attractive therapeutic targets of the disease^20,21^.

The first study to characterize lncRNAs genome-wide in OA cartilage was performed by Ajekigbe *et al*.^22^, who performed an RNA-Seq assay in samples from hip cartilage of ten patients with OA and six control subjects who had a neck-of-femur fracture. One hundred ninety-eight lncRNAs were found to be significantly differentially expressed between OA and healthy hip cartilage. The authors subsequently compared the obtained lncRNAs with those from a knee dataset, concluding that 1834 lncRNAs were expressed in both hip and knee cartilages. Of the 198 and 93 differentially expressed hip and knee lncRNAs (respectively), only 20, including both NEAT1 and MEG3, were common to the two cartilages^22^. Interestingly, lncRNA MEG3 has been described to not only be involved in osteogenic differentiation and bone diseases^23^ but also contribute to cell proliferation and inhibit apoptosis and extracellular matrix degradation via the miR-361-5p/*FOXO1* axis in OA chondrocytes^24^. These findings led investigators to consider this lncRNA as an attractive therapeutic target in OA.

lncRNA MFI2-AS1 could be considered another therapeutic target in OA. The expression of this lncRNA is increased in OA tissues and in lipopolysaccharide-treated C28/I2 cells; however, knockdown of MFI2-AS1 suppresses apoptosis, inflammatory response, and extracellular matrix degradation by increasing miR-130a-3p^25^.

## Other small non-coding RNAs

Research into the role of other types of non-coding RNAs in OA is starting to take off. This is the case of circular RNAs (circRNAs) mainly, but also both small nucleolar RNAs (snoRNAs) and transferent RNA-derived fragments (tRFs) stand out.

CircRNAs are a class of non-coding RNAs that circularize as a covalently closed loop structure without a 5′-cap structure and 3′-poly A tail. They are able to regulate gene expression by sponging RNA-binding proteins and miRNAs. Recent studies describe a series of functions of circRNAs in OA, including the modulation of metabolic homeostasis in cartilage matrix as well as their participation in inflammation, chondrocyte apoptosis, proliferation, and autophagy^26,27^. Examples of some recently discovered circRNAs as biomarkers in OA include the hsa_circ_0032132, which is significantly increased in the blood levels of patients with OA compared with healthy controls^28^, and the hsa_circ_0101873, hsa_circ_0104595, and hsa_circ_0101251, whose levels are significantly increased in the synovial fluid of patients with OA and even show a positive correlation with radiographic severity and WOMAC (Western Ontario and McMaster Universities Arthritis Index) score^29^. Similarly, hsa_circ_101178 has been proposed as a biomarker for early diagnosis of OA since the serum levels of this circRNA are significantly higher in patients with OA and, in addition, these levels show a significant positive correlation with the severity of the disease in terms of KL grade and WOMAC pain score^30^. The number of studies involving circRNAs in OA has increased dramatically in recent years, but an in-depth description of both the biological roles and the mechanisms of action of these small non-coding RNAs is beyond the scope of this review.

SnoRNAs are non-coding RNAs located in the nucleolus and are involved in ribosomal RNA (rRNA) modifications and therefore are key elements for the production of efficient RNAs. Most snoRNAs are necessary for biological processes such as mRNA splicing, genome stability, or protein translation, and they are responsible for the modification of other non-coding RNAs. In OA, the first microarray study using articular cartilage from young, old, and OA knees identified panels of snoRNAs differentially expressed, including SNORD96A as heavily reduced with age and SNORD26 as significantly overexpressed in OA^31^. The authors demonstrated that the alteration of different snoRNAs affected chondrogenic and hypertrophic gene expression and these snoRNAs could serve as therapeutic targets in OA^31^.

tRFs are key small non-coding RNAs that regulate gene expression on multiple levels, including translation, transcription, apoptosis, cell proliferation, and ribosome biogenesis. Aberrant expression of these non-coding RNAs was detected in different human diseases, including cancer and neurodegenerative disorders^32^. In OA, the knowledge of tRFs is still scarce; however, their study opens a new area of their potential use as therapeutic targets and biomarkers in OA. Even though Zacharjasz *et al*. published a summary of differentially expressed tRFs in OA, only one study to date aimed to determine the function of the most upregulated tRF in OA, tRNA-Cys-GCA in interleukin 1 beta (*IL-1β*)-stimulated chondrocytes. The authors found that this tRF regulates the Janus kinase 3 (*JAK3*) expression in chondrocytes, therefore regulating the expression of different cytokines, including *IL-6*^33^.

## DNA methylation

Among epigenetic modifications, DNA methylation is the best characterized and consists of the addition of a methyl group to DNA at specific CpG sites and generally correlates with gene silencing. This epigenetic modification in OA has been the focus of different studies in recent years. In fact, it has been described that a large number of genetic risk variants associated with OA correlate with DNA methylation changes that take place in cartilage^34^. The first methylation studies consisted of the analysis of specific CpG sites in the promoter regions of genes involved in the development of OA, such as metalloproteinases^35^. However, in recent years, the use of genome-wide arrays emerged and specific methylation profiles have been described in knee and hip cartilage^36,37^. These studies identified specific clusters of OA patients with altered methylation patterns in genes involved in the inflammatory response. A more recent study, performed in peripheral blood mononuclear cells, developed methylation models to predict radiographic progression^38^. Despite the relatively small sample size, their models including methylation data were significantly more predictive of radiographic progression than those models including only patient characteristics, and a set of associated genes and pathways, some of them previously associated with OA development, has been proposed to be associated with rapid OA progression^38^.

Of special interest is the analysis of methylation dynamics in enhancers. Enhancers are regulatory DNA sequences that, when bound by transcription factors, stimulate (enhance) the transcription of an associated gene or genes. Therefore, these sequences have pivotal roles in the regulation of gene expression. An interesting study by Xiaozong Lin *et al*.^39^ explored the methylation dynamics in enhancers by using a public dataset from a study by Rushton *et al*.^37^, performed on 108 cartilage samples from patients with knee OA and hip OA and also hip cartilages from healthy donors. After analyzing the data, Xiaozong Lin *et al*. observed that enhancers suffer major methylation changes in comparison to other genetic regions. The authors also identified a huge percentage of differentially methylated CpG in enhancers between male and female patients with hip OA, most of them significantly hypomethylated in females, as well as organ source–dependent differences in enhancer methylation between hip and knee OA. In summary, the authors conclude that their enhancer methylation atlas can be used as a biomarker of OA subtypes^39^.

Another interesting bioinformatics approach combined three different public datasets: two expression profiles and one methylation data. The authors identified 133 abnormally methylated and differentially expressed genes, of which 85 were hypomethylated and correlated with high-expression genes and 48 were hypermethylated and correlated with low-expression genes. Subsequent combination of protein–protein interaction (PPI) network construction and co-expression analysis revealed that hypomethylated and high-expression genes *COL3A1*, *LUM*, and *MMP2* are hub genes for end-stage OA, and the authors propose *THBS2* as a novel biomarker for end-stage OA too^40^.

Methylation quantitative trait loci (mQTLs) are genetic variants that correlate with methylation levels at a CpG site. Up to 25% of OA risk loci are considered mQTLs^34^. Rice *et al*. combined genotyping, DNA methylation, and RNA-Seq data from the cartilage of patients who had undergone arthroplasty with *in silico* analyses to investigate 42 OA risk loci. In 10 of these 42 risk loci, 24 CpGs in which methylation correlated with genotype were identified. The *in silico* analyses of these 10 mQTLs revealed collagen beta (1-O) galactosyltransferase 2 (*COLGALT2*), collagen 11 type A 2 (*COL11A2*), and WW domain-containing E3 ubiquitin protein ligase 2 (*WWP2*) as emerging key target genes^41^. Interestingly, mitochondrial DNA (mtDNA) variations seem to act as mQTLs too. Cortés-Pereira *et al*. demonstrated a differential association of mtDNA haplogroups H and J with the methylation status of articular cartilage. The differential expression of nuclear genes affects important processes involved in OA, such as apoptosis and dysregulated metabolism and development^42^. mtDNA haplogroups H and J have antagonistic effects in the evolution of OA^43^, and their impact on the pathogenesis of OA could be mediated by the retrograde control that mitochondria exert over the nucleus through a series of signaling mechanisms that alter DNA methylation pattern^42^. Therefore, since the epigenetic modifications that take place in OA differ between haplogroups, these mtDNA variants could be considered complementary genetic biomarkers of the disease.

## Genome-wide association studies

In recent years, GWASs became important genetic tools that permitted investigators to understand the polygenic nature of OA. To date, up to 124 single-nucleotide polymorphisms (SNPs) encompassing 95 independent loci have been associated with OA^34^. The effect size of risk loci for OA, as for many common polygenic diseases but not for Mendelian diseases, is quite small and therefore the “liability threshold” model can be applied to OA. This model considers that the higher the number of risk variants an individual harbors beyond a threshold number, the higher the susceptibility to develop the disease^44^. Another interesting aspect is the fact that, as in many other common diseases, most of the GWAS signals have been reported in non-coding regions of the genome^34^.

The complex post-GWAS analyses led to the identification of target genes of OA-associated SNPs as well as genes that have a therapeutic approved or in clinical trials. Among the target genes, *COLGALT2*, *RUNX2*, *PLEC*, *MGP*, *ALDH1A2*, *TGFB1*, *GDF5*, *RWDD2B*, *CDC5L*, and *SMAD3* stand out. Interestingly, the coded proteins of these genes are involved in biological functions related to OA, such as cytoskeletal protein (*PLEC*), extracellular signaling (*GDF5* and *TGFB1*), transcription factor essential for osteoblastic differentiation and skeletogenesis (*RUNX2*), intracellular enzyme (*ALDH1A2*), or extracellular calcium regulator (*MGP*)^34^. Of these, *TGFB1* and *GDF5*, together with *FGF18*, *CTSK*, *IL11*, *DPEP1*, *DIABLO*, *CRHR1*, *MAPT*, and *TNFSF15*, have a therapeutic approved or in clinical trials^45^.

## Mitochondrial genetics

Mitochondria are cellular organelles whose main function is the production of ATP through the oxidative phosphorylation system; however, they have been implicated in other cellular processes such as calcium homeostasis, apoptosis signaling, redox balance, or inflammation. Impaired mitochondria leads to a series of events—such as increased apoptosis, inflammatory, and catabolic response as well as a decrease of chondrocyte anabolic and growth response—that contribute to the development of OA^43^. In addition, mitochondria contain their own genetic material, mtDNA, with specific stable genetic polymorphisms inherited through the maternal line as a consequence of the adaptations that permitted humans to adapt to different climates^46^. These genetic polymorphisms are called haplogroups and have been associated with the risk of different human diseases, including OA^47,48^.

In recent years, mtDNA haplogroups have been consolidated as biomarkers of prevalence, incidence, and progression of OA in Caucasian and non-Caucasian populations. Results of different works demonstrated that subjects carrying the mtDNA haplogroup J show a lower risk of OA susceptibility^49^ as well as a lower risk of knee OA incidence when compared with the haplogroup H^50^. In addition, patients with the mtDNA cluster TJ show a lower risk of knee OA progression when compared with patients in the mtDNA cluster HV^51^. Mechanisms that could explain these findings include a lower rate of apoptosis in addition to lower levels of reactive oxygen species (ROS) and higher survival under oxidative stress conditions in cells harboring these haplogroups^50^. Similar results were obtained in non-Caucasian populations. Specifically, haplogroup G predisposes to an increased risk of OA in southern China populations because of lower glycolysis activity^52^. In addition, haplogroup B is associated with an increased incidence of knee OA in Koreans^53^.

Different studies in animal models of conplastic mice support the above-mentioned findings. Conplastic mice consist of mice with the same nuclear genome but different mtDNA variants. On the one hand, these studies showed that mtDNA haplotype profoundly influenced ROS generation and mitochondrial proteostasis as well as insulin signaling, obesity, telomere shortening, and mitochondrial dysfunction^54^. In the field of OA, the use of these animals demonstrated the functional impact of non-pathological variants of mtDNA on OA process using a surgically induced OA model. The latter study demonstrated that specific conplastic mice BL/6^NZB^ developed less severe OA, as indicated by a reduced Osteoarthritis Research Society International (OARSI) histopathology score and synovitis, accompanied by higher autophagy and lower apoptosis^55^.

## Conclusions and future directions

Some of the most relevant genetic biomarkers in OA are shown in Table 1. On the one hand, the amount of genomic data available so far permitted investigators to identify a series of biological processes that, to a greater or lesser extent, could explain the impact of genetic variants derived from GWASs on the development of the disease as well as related proteins involved in target therapeutic pathways. Among these processes, articular joint maintenance, homeostasis, and formation stand out^34^. However, given the polygenic nature of OA and the fact that the proportion of heritability for the currently known risk loci is just 22.5%^45^, the remaining unknown loci are still large. On the other hand, the development of high-quality single-cell sequencing led to the identification of novel phenotypes of chondrocytes and synoviocytes, which could serve as targets for early therapeutic interventions in OA. In addition, among the huge number of non-coding RNAs involved in the development of OA, some of them appear to be not only robust candidate diagnostic biomarkers but also therapeutic targets of the disease.

**Table 1.** Relevant genetic biomarkers in osteoarthritis.

Genomic approach	Genomic region	Outcome	References
Single-cell RNA sequencing	*COL6A3*, *ACTG1*	Progression biomarkers	3
microRNAs	miR-99-3p	Diagnostic biomarker	9
	miR-34a-5p	Late knee OA biomarker in obese patients	10
	hsa-miR-335-3p, hsa-miR-199a-5p, hsa-miR- 671-3p, hsa-miR-1260b, hsa-miR-191-3p, hsa-miR-335-5p	Early symptomatic knee OA biomarkers	11
	miR-146a-5p miR-186-5p	Prevalent and incident knee OA in women	12
	miR-let-7e	Severity biomarker, defective autophagy	6,17
Long non-coding RNAs	lncRNA NEAT1 lncRNA MEG3	Diagnostic biomarkers/Therapeutic targets	22,24
	lncRNA MFI2-AS1	Diagnostic biomarkers/ Therapeutic targets	25
Circular RNAs	hsa_circ_0032132	Diagnostic biomarker	28
	hsa_circ_0101873	Diagnostic/Severity biomarkers	29
	hsa_circ_0104595		
	hsa_circ_0101251		
	hsa_circ_101178	Early symptomatic knee OA biomarker	30
Small nucleolar RNAs	SNORD96A	Aging biomarker	31
	SNORD26	Diagnostic biomarker/Therapeutic target	
Transferent RNA-derived fragments	tRNA-Cys-GCA	Diagnostic biomarker/Therapeutic target	33
DNA methylation	*COL3A1*, *LUM*, *MMP2*, *THBS2*	End-stage OA biomarkers	40
	*COLGALT2*, *COL11A2*, *WWP2*	End-stage OA biomarkers	41
Genome-wide association study	*COLGALT2*, *RUNX2, PLEC, MGP, ALDH1A2,* *TGB1, GDF5, RWDD2B, CDC5L, SMAD3*	Target genes associated with the development of OA	34
	*TGB1, GDF5, FGF18, CTSK, IL11, DPEP1,* *DIABLO, CRHR1, MAPT, TNFSF15*	Therapeutic approved or in clinical trials	45
Mitochondrial genetics	Haplogroup J	Prevalence and incidence knee OA biomarker in Caucasian populations	49,50
	mtDNA cluster JT	Knee OA progression biomarker in Caucasian populations	51
	Haplogroup G	Prevalence knee OA biomarker in Chinese	52
	Haplogroup B	Incidence knee OA biomarker in Koreans	53

*ACTG1*, actin gamma 1; *ALDH1A2*, aldehyde dehydrogenase 1 family member A2; *CDC5L*, cell division cycle 5 like; *COL3A1*, collagen type III alpha 1 chain; *COL6A3*, collagen type VI alpha 3 chain; *COLGALT2*, collagen beta(1-O)galactosyltransferase 2; *COL11A2*, collagen type XI alpha 2 chain; *CRHR1*, corticotropin releasing hormone receptor 1; *CTSK*, cathepsin K; *DIABLO*, diablo IAP-binding mitochondrial protein; *DPEP1*, dipeptidase 1; *FGF18*, fibroblast growth factor 18; *GDF5*, growth differentiation factor 5; hsa-miR, human microRNA; *IL11*, interleukin 11; lncRNA, long non-coding RNA; *LUM*, lumican; *MAPT*, microtubule associated protein tau; MEG3, maternally expressed 3; *MGP*, matrix Gla protein; *MMP2*, matrix metallopeptidase 2; mtDNA, mitochondrial DNA; NEAT1, nuclear paraspeckle assembly transcript 1; OA, osteoarthritis; *PLEC*, plectin; *RUNX2*, RUNX family transcription factor 2; *RWDD2B*, RWD domain-containing 2B; *SMAD3*, SMAD family member 3; *TGB1*, transforming growth factor beta 1; *THBS2*, thrombospondin 2; *TNFSF15*, transforming growth factor superfamily member 15; *WWP2*, WW domain-containing E3 ubiquitin protein ligase 2

Future approaches should focus on the discovery of the largely still unknown OA-related loci and try to find their functional significance in order to design a diagnostic tool that permits the early identification of OA phenotypes. In addition, the complete characterization of the OA-related proteins and the understanding of the different pathways involved in the development of OA should serve to elaborate adequate therapeutic strategies to design effective drugs to treat this disease.

## References

[1] Gawad C Koh W Quake SR : Single-cell genome sequencing: Current state of the science. *Nat Rev Genet.* 2016; 17(3): 175–88. 10.1038/nrg.2015.16 26806412

[2] Ji Q Zheng Y Zhang G : Single-cell RNA-seq analysis reveals the progression of human osteoarthritis. *Ann Rheum Dis.* 2019; 78(1): 100–10. 10.1136/annrheumdis-2017-212863 30026257 PMC6317448

[3] Li C Luo J Xu X : Single cell sequencing revealed the underlying pathogenesis of the development of osteoarthritis. *Gene.* 2020; 757: 144939. 10.1016/j.gene.2020.144939 32640306

[4] Chou CH Jain V Gibson J : Synovial cell cross-talk with cartilage plays a major role in the pathogenesis of osteoarthritis. *Sci Rep.* 2020; 10(1): 1087. 10.1038/s41598-020-67730-y 32616761 PMC7331607

[5] Lagos-Quintana M Rauhut R Lendeckel W : Identification of novel genes coding for small expressed RNAs. *Science.* 2001; 294(5543): 853–8. 10.1126/science.1064921 11679670

[6] Beyer C Zampetaki A Lin NY : Signature of circulating microRNAs in osteoarthritis. *Ann Rheum Dis.* 2015; 74(3): e18. 10.1136/annrheumdis-2013-204698 24515954

[7] Díaz-Prado S Cicione C Muiños-López E : Characterization of microRNA expression profiles in normal and osteoarthritic human chondrocytes. *BMC Musculoskelet Disord.* 2012; 13: 483. 10.1186/1471-2474-13-144 22883423 PMC3495209

[8] Endisha H Rockel J Jurisica I : The complex landscape of microRNAs in articular cartilage: Biology, pathology, and therapeutic targets. *JCI Insight.* 2018; 3(17): e121630. 10.1172/jci.insight.121630 30185670 PMC6171796

[9] Coutinho de Almeida R Ramos YFM Mahfouz A : RNA sequencing data integration reveals an miRNA interactome of osteoarthritis cartilage. *Ann Rheum Dis.* 2019; 78(2): 270–7. 10.1136/annrheumdis-2018-213882 30504444 PMC6352405

[10] Endisha H Datta P Sharma A : MicroRNA-34a-5p Promotes Joint Destruction During Osteoarthritis. *Arthritis Rheumatol.* 2021; 73(3): 426–39. 10.1002/art.41552 33034147 PMC7986901

[11] Ali SA Gandhi R Potla P : Sequencing identifies a distinct signature of circulating microRNAs in early radiographic knee osteoarthritis. *Osteoarthritis Cartilage.* 2020; 28(11): 1471–81. 10.1016/j.joca.2020.07.003 32738291

[12] Rousseau JC Millet M Croset M : Association of circulating microRNAs with prevalent and incident knee osteoarthritis in women: The OFELY study. *Arthritis Res Ther.* 2020; 22(1): 2. 10.1186/s13075-019-2086-5 31898522 PMC6941326

[13] Caramés B Taniguchi N Otsuki S : Autophagy is a protective mechanism in normal cartilage, and its aging-related loss is linked with cell death and osteoarthritis. *Arthritis Rheum.* 2010; 62(3): 791–801. 10.1002/art.27305 20187128 PMC2838960

[14] Li H Li Z Pi Y : MicroRNA-375 exacerbates knee osteoarthritis through repressing chondrocyte autophagy by targeting ATG2B. *Aging (Albany NY).* 2020; 12(8): 7248–61. 10.18632/aging.103073 32335541 PMC7202526

[15] Zhao X Li H Wang L : MicroRNA-107 regulates autophagy and apoptosis of osteoarthritis chondrocytes by targeting TRAF3. *Int Immunopharmacol.* 2019; 71: 181–7. 10.1016/j.intimp.2019.03.005 30909133

[16] Zhong G Long H Ma S : miRNA-335-5p relieves chondrocyte inflammation by activating autophagy in osteoarthritis. *Life Sci.* 2019; 226: 164–72. 10.1016/j.lfs.2019.03.071 30970265

[17] Feng L Feng C Wang CX : Circulating microRNA let-7e is decreased in knee osteoarthritis, accompanied by elevated apoptosis and reduced autophagy. *Int J Mol Med.* 2020; 45(5): 1464–1476. 10.3892/ijmm.2020.4534 32323821 PMC7138275

[18] Robinson EK Covarrubias S Carpenter S : The how and why of lncRNA function: An innate immune perspective. *Biochim Biophys Acta Gene Regul Mech.* 2020; 1863(4): 194419. 10.1016/j.bbagrm.2019.194419 31487549 PMC7185634

[19] Yao RW Wang Y Chen LL : Cellular functions of long noncoding RNAs. *Nat Cell Biol.* 2019; 21(5): 542–51. 10.1038/s41556-019-0311-8 31048766

[20] Tu J Huang W Zhang W : The emerging role of lncRNAs in chondrocytes from osteoarthritis patients. *Biomed Pharmacother.* 2020; 131: 110642. 10.1016/j.biopha.2020.110642 32927251

[21] Xie F Liu YL Chen XY : Role of MicroRNA, LncRNA, and Exosomes in the Progression of Osteoarthritis: A Review of Recent Literature. *Orthop Surg.* 2020; 12(3): 708–16. 10.1111/os.12690 32436304 PMC7307224

[22] Ajekigbe B Cheung K Xu Y : Identification of long non-coding RNAs expressed in knee and hip osteoarthritic cartilage. *Osteoarthritis Cartilage.* 2019; 27(4): 694–702. 10.1016/j.joca.2018.12.015 30611906 PMC6444060

[23] Sun H Peng G Wu H : Long non-coding RNA MEG3 is involved in osteogenic differentiation and bone diseases (Review). *Biomed Rep.* 2020; 13(1): 15–21. 10.3892/br.2020.1305 32494359 PMC7257936

[24] Wang A Hu N Zhang Y : MEG3 promotes proliferation and inhibits apoptosis in osteoarthritis chondrocytes by miR-361-5p/FOXO1 axis. *BMC Med Genomics.* 2019; 12(1): 201. 10.1186/s12920-019-0649-6 31888661 PMC6937924

[25] Luo X Wang J Wei X : Knockdown of lncRNA MFI2-AS1 inhibits lipopolysaccharide-induced osteoarthritis progression by miR-130a-3p/TCF4. *Life Sci.* 2020; 240: 117019. 10.1016/j.lfs.2019.117019 31678554

[26] Liu D Liang YH Yang YT : Circular RNA in osteoarthritis: An updated insight into the pathophysiology and therapeutics. *Am J Transl Res.* 2021; 13(1): 11–23. 33527005 PMC7847522

[27] Mao X Cao Y Guo Z : Biological roles and therapeutic potential of circular RNAs in osteoarthritis. *Mol Ther Nucleic Acids.* 2021; 24: 856–67. 10.1016/j.omtn.2021.04.006 34026329 PMC8131397

[28] Wang Y Wu C Zhang Y : Screening for differentially expressed circRNA between Kashin-Beck disease and osteoarthritis patients based on circRNA chips. *Clin Chim Acta.* 2020; 501: 92–101. 10.1016/j.cca.2019.10.026 31678276

[29] Yu F Xie C Sun J : Circular RNA expression profiles in synovial fluid: A promising new class of diagnostic biomarkers for osteoarthritis. *Int J Clin Exp Pathol.* 2018; 11(3): 1338–46. 31938229 PMC6958132

[30] Chen C : Serum hsa_circ_101178 as a Potential Biomarker for Early Prediction of Osteoarthritis. *Clin Lab.* 2020; 66(8). 10.7754/Clin.Lab.2020.191251 32776746

[31] Peffers MJ Chabronova A Balaskas P : SnoRNA signatures in cartilage ageing and osteoarthritis. *Sci Rep.* 2020; 10(1): 10641. 10.1038/s41598-020-67446-z 32606371 PMC7326970

[32] Zacharjasz J Mleczko AM Bąkowski P : Small Noncoding RNAs in Knee Osteoarthritis: The Role of MicroRNAs and tRNA-Derived Fragments. *Int J Mol Sci.* 2021; 22(11): 5711. 10.3390/ijms22115711 34071929 PMC8198041

[33] Green JA Ansari MY Ball HC : tRNA-derived fragments (tRFs) regulate post-transcriptional gene expression via AGO-dependent mechanism in IL-1β stimulated chondrocytes. *Osteoarthritis Cartilage.* 2020; 28(8): 1102–10. 10.1016/j.joca.2020.04.014 32407895 PMC8418333

[34] Aubourg G Rice SJ Bruce-Wootton P : Genetics of osteoarthritis. *Osteoarthritis Cartilage.* 2021; S1063-4584(21)00632-4. 10.1016/j.joca.2021.03.002 33722698 PMC9067452

[35] Roach HI Yamada N Cheung KSC : Association between the abnormal expression of matrix-degrading enzymes by human osteoarthritic chondrocytes and demethylation of specific CpG sites in the promoter regions. *Arthritis Rheum.* 2005; 52(10): 3110–24. 10.1002/art.21300 16200590

[36] Fernández-Tajes J Soto-Hermida A Vázquez-Mosquera ME : Genome-wide DNA methylation analysis of articular chondrocytes reveals a cluster of osteoarthritic patients. *Ann Rheum Dis.* 2014; 73(4): 668–77. 10.1136/annrheumdis-2012-202783 23505229

[37] Rushton MD Reynard LN Barter MJ : Characterization of the cartilage DNA methylome in knee and hip osteoarthritis. *Arthritis Rheumatol.* 2014; 66(9): 2450–60. 10.1002/art.38713 24838673 PMC4314681

[38] M Dunn C Nevitt MC Lynch JA : A pilot study of peripheral blood DNA methylation models as predictors of knee osteoarthritis radiographic progression: Data from the Osteoarthritis Initiative (OAI). *Sci Rep.* 2019; 9(1): 16880. 10.1038/s41598-019-53298-9 31727952 PMC6856188

[39] Lin X Li L Liu X : Genome-wide analysis of aberrant methylation of enhancer DNA in human osteoarthritis. *BMC Med Genomics.* 2020; 13(1): 1. 10.1186/s12920-019-0646-9 31900157 PMC6942377

[40] Zheng L Chen W Xian G : Identification of abnormally methylated-differentially expressed genes and pathways in osteoarthritis: A comprehensive bioinformatic study. *Clin Rheumatol.* 2021; 40(8): 3247–56. 10.1007/s10067-020-05539-w 33420869

[41] Rice SJ Cheung K Reynard LN : Discovery and analysis of methylation quantitative trait loci (mQTLs) mapping to novel osteoarthritis genetic risk signals. *Osteoarthritis Cartilage.* 2019; 27(10): 1545–56. 10.1016/j.joca.2019.05.017 31173883

[42] Cortés-Pereira E Fernández-Tajes J Fernández-Moreno M : Differential Association of Mitochondrial DNA Haplogroups J and H With the Methylation Status of Articular Cartilage: Potential Role in Apoptosis and Metabolic and Developmental Processes. *Arthritis Rheumatol.* 2019; 71(7): 1191–200. 10.1002/art.40857 30747498

[43] Blanco FJ Valdes AM Rego-Pérez I : Mitochondrial DNA variation and the pathogenesis of osteoarthritis phenotypes. *Nat Rev Rheumatol.* 2018; 14(6): 327–40. 10.1038/s41584-018-0001-0 29670212

[44] Dempster ER Lerner IM : Heritability of threshold characters. *Genetics.* 1950; 35(2): 212–36. 10.1093/genetics/35.2.212 17247344 PMC1209482

[45] Tachmazidou I Hatzikotoulas K Southam L : Identification of new therapeutic targets for osteoarthritis through genome-wide analyses of UK Biobank data. *Nat Genet.* 2019; 51(2): 230–6. 10.1038/s41588-018-0327-1 30664745 PMC6400267

[46] Ruiz-Pesini E Mishmar D Brandon M : Effects of purifying and adaptive selection on regional variation in human mtDNA. *Science.* 2004; 303(5655): 223–6. 10.1126/science.1088434 14716012

[47] Wallace DC Brown MD Lott MT : Mitochondrial DNA variation in human evolution and disease. *Gene.* 1999; 238(1): 211–30. 10.1016/s0378-1119(99)00295-4 10570998

[48] Zhao Z Li Y Wang M : Mitochondrial DNA haplogroups participate in osteoarthritis: Current evidence based on a meta-analysis. *Clin Rheumatol.* 2020; 39(4): 1027–37. 10.1007/s10067-019-04890-x 31897963

[49] Rego-Pérez I Fernández-Moreno M Fernández-López C : Mitochondrial DNA haplogroups: Role in the prevalence and severity of knee osteoarthritis. *Arthritis Rheum.* 2008; 58(8): 2387–96. 10.1002/art.23659 18668590

[50] Fernández-Moreno M Soto-Hermida A Vázquez-Mosquera ME : Mitochondrial DNA haplogroups influence the risk of incident knee osteoarthritis in OAI and CHECK cohorts. A meta-analysis and functional study. *Ann Rheum Dis.* 2017; 76(6): 1114–22. 10.1136/annrheumdis-2016-210131 27919866

[51] Fernández-Moreno M Soto-Hermida A Vázquez-Mosquera ME : A replication study and meta-analysis of mitochondrial DNA variants in the radiographic progression of knee osteoarthritis. *Rheumatology (Oxford).* 2017; 56(2): 263–70. 10.1093/rheumatology/kew394 27864563 PMC5854036

[52] Fang H Zhang F Li F : Mitochondrial DNA haplogroups modify the risk of osteoarthritis by altering mitochondrial function and intracellular mitochondrial signals. *Biochim Biophys Acta.* 2016; 1862(4): 829–36. 10.1016/j.bbadis.2015.12.017 26705675

[53] Koo BS Song Y Lee S : Association of Asian mitochondrial DNA haplogroup B with new development of knee osteoarthritis in Koreans. *Int J Rheum Dis.* 2019; 22(3): 411–6. 10.1111/1756-185X.13453 30549233

[54] Latorre-Pellicer A Moreno-Loshuertos R Lechuga-Vieco AV : Mitochondrial and nuclear DNA matching shapes metabolism and healthy ageing. *Nature.* 2016; 535(7613): 561–5. 10.1038/nature18618 27383793

[55] Scotece M Rego-Pérez I Lechuga-Vieco AV : Mitochondrial DNA impact on joint damaged process in a conplastic mouse model after being surgically induced with osteoarthritis. *Sci Rep.* 2021; 11(1): 9112. 10.1038/s41598-021-88083-0 33907208 PMC8079696

